# Intervention development for integration of conventional tobacco cessation interventions into routine CAM practice

**DOI:** 10.1186/s12906-015-0604-9

**Published:** 2015-03-29

**Authors:** Myra L Muramoto, Eva Matthews, Cheryl K Ritenbaugh, Mark A Nichter

**Affiliations:** Department of Family and Community Medicine, University of Arizona College of Medicine, P.O. Box 245052, Tucson, AZ 85724 USA; University of Arizona, School of Anthropology, 1009 E. South Campus Drive, Tucson, AZ 85721 USA

**Keywords:** Context validity, Intervention protocol, Curriculum development, Training, Interprofessional education, Tobacco cessation, Chiropractic, Acupuncture, Massage therapist, Community based participatory research

## Abstract

**Background:**

Practitioners of complementary and alternative medicine (CAM) therapies are an important and growing presence in health care systems worldwide. A central question is whether evidence-based behavior change interventions routinely employed in conventional health care could also be integrated into CAM practice to address public health priorities. Essential for successful integration are intervention approaches deemed acceptable and consistent with practice patterns and treatment approaches of different types of CAM practitioners – that is, they have *context validity*. Intervention development to ensure context validity was integral to Project CAM Reach (CAMR), a project examining the public health potential of tobacco cessation training for chiropractors, acupuncturists and massage therapists (CAM practitioners). This paper describes formative research conducted to achieve this goal.

**Methods:**

Intervention development, undertaken in three CAM disciplines (chiropractic, acupuncture, massage therapy), consisted of six iterative steps: 1) exploratory key informant interviews; 2) local CAM practitioner community survey; 3) existing tobacco cessation curriculum demonstration with CAM practitioners; 4) adapting/tailoring of existing curriculum; 5) external review of adaptations; 6) delivery of tailored curriculum to CAM practitioners with follow-up curriculum evaluation.

**Results:**

CAM practitioners identified barriers and facilitators to addressing tobacco use with patients/clients and saw the relevance and acceptability of the intervention content. The intervention development process was attentive to their real world intervention concerns. Extensive intervention tailoring to the context of each CAM discipline was found unnecessary. Participants and advisors from all CAM disciplines embraced training content, deeming it to have broad relevance and application across the three CAM disciplines. All findings informed the final intervention.

**Conclusions:**

The participatory and iterative formative research process yielded an intervention with context validity in real-world CAM practices as it: 1) is patient/client-centered, emphasizing the practitioner’s role in a healing relationship; 2) is responsive to the different contexts of CAM practitioners’ work and patient/client relationships; 3) integrates relevant best practices from US Public Health Service Clinical Practice Guidelines on treating tobacco dependence; and 4) is suited to the range of healing philosophies, scopes of practice and practice patterns found in participating CAM practitioners. The full CAMR study to evaluate the impact of the CAMR intervention on CAM practitioners’ clinical behavior is underway.

## Background

Complementary and alternative medicine (CAM) therapy use is an increasingly important factor in the health care landscape [1]. A number of national surveys indicate substantial [2-6] and in some cases growing use [7] of CAM therapies. In the United States (U.S.), between the 2002 and 2007 National Health Interview Survey (NHIS), the proportion of adults reporting use of at least one form of CAM increased from 25.7% to 29.4% - a relative increase of 14.2%. Provider-based CAM (pbCAM, including all those services which require the presence of a practitioner as contrasted with CAM treatments that can be self-administered, such as herbal medicines) use saw a relative increase of 29.6%. The most significant increases in pbCAM use were seen in chiropractic care, acupuncture, massage therapy, and folk medicine [7]. Harris and colleagues [6] note in their international review of CAM use studies that more population-based assessment (i.e. through government sponsored health surveys) of CAM use is necessary to provide a more accurate picture of trends in prevalence of CAM use over time.

### Context validity of interventions in CAM practices

Growth in CAM use has inspired innovative attempts to offer patients holistic care through integrating CAM into conventional medical practice [8-10]. By contrast, there has been much less exploration of how interventions widely used in conventional medicine and behavioral health might be effectively incorporated into pbCAM practice as a means of advancing the preventive and promotive health goals of both CAM and conventional medicine [11]. CAM practitioners and practices differ markedly from conventional medicine practitioners and practices with respect to professional training, practice patterns, business models, treatment and healing paradigms, philosophical orientation to the patient/client – practitioner relationship and perceptions of treatment effectiveness [11-18]. A central question that arises is whether evidence-based health care and/or behavior change interventions widely employed in conventional medical and behavioral health care could be integrated into the CAM practice context in ways that ensure validity within the specific context of a CAM practice while maintaining the conceptual integrity of the evidence-based intervention. In this paper we refer to this concept as *“context validity”.* Intervention development to ensure context validity requires addressing such questions as: Will these interventions be acceptable to practitioners in different CAM disciplines? Do the interventions “fit” or make sense within the training and healing traditions, scope of practice, and practice patterns relevant to practitioners of the specific CAM disciplines who would be asked to implement the intervention? In order for integration to be effective, interventions would at once need to be tailored into real world CAM practices; yet maintain their conceptual integrity and be subject to established evaluation criteria.

### Project CAM Reach

Context validity of the research intervention is a key aspect of Project CAM Reach (CAMR), a National Cancer Institute (NCI) sponsored study examining the public health potential of tobacco cessation training for chiropractors, acupuncturists and massage therapists (CAM practitioners). The CAMR study has two main aims. First, develop an intervention protocol, a tobacco cessation brief intervention training and practice-system intervention that includes appropriate tobacco cessation best practices from the U.S. Public Health Service Guideline on Treatment of Tobacco Dependence (PHS Guideline) [19] and is tailored for the needs of CAM practitioners. Second, in the real world of CAM practices, evaluate the impact of the CAMR intervention on CAM practitioners’ knowledge, attitudes and practice behaviors with respect to integration of tobacco cessation practices recommended by the PHS guideline [19].

The inspiration for CAMR is three-fold. First, the growing burden of chronic disease is at the heart of the US health care crisis. Chronic disease accounts for more than 75% of health care costs in the US and the steady escalation of the nation’s health care bill is driven in large part by the increasing costs of caring for chronic disease [20-22]. Globally, chronic diseases are the largest cause of death. The leading chronic diseases share common life-style related major risk factors of tobacco use, unhealthy diet, physical inactivity, and alcohol use [23,24]. Second, CAM practitioners have characteristics and practice patterns that make them well suited to addressing lifestyle-related chronic disease risk factors. Third, local CAM practitioners participating in a tobacco-cessation training project for lay community members (described below) requested that tobacco cessation training be made more available to their disciplines [25].

### Tobacco cessation and CAM practitioners

Even after decades of public health tobacco control efforts, tobacco remains the single largest preventable cause of death globally [26]. In the U.S., where the current work was conducted, tobacco cessation brief interventions (BIs) based on the 5A’s framework (Ask, Advise, Assess, Assist, Arrange) [27], and that also include intra-treatment social support, continue to form the backbone of practice-based conventional healthcare intervention. More recently, BIs are being evaluated in developing nations [28,29]. That said, despite clear evidence from the U.S. that BIs by health care providers result in increased tobacco cessation rates [19], and that such BIs are the most cost-effective preventive health services [30], implementation of BIs by biomedical physicians fall far short of the ideal [31]. For nearly 3 decades, cessation training in the US has focused on conventional biomedical health practitioners, primarily physicians. Only more recently has cessation training included non-physicians, e.g. nurses, respiratory therapists, dentists, and dental hygienists [27,32]. But with rare exceptions [33], the focus remains on training biomedical health professionals.

CAM practitioners have characteristics and practice patterns that may make them better suited to health and wellness promotion than conventional practitioners. Compared to conventional biomedical practitioners, visits with CAM practitioners are often longer and more frequent [13,34,35], providing more time to address complex lifestyle issues. They often see patients for regular health maintenance/wellness care, allowing for repeated follow-ups and reassessment of behavioral changes [13].

Analysis of 2002 and 2007 data from the National Health Interview Survey in the U.S. found that CAM practitioners provide care for significant numbers of smokers [36]. A population-based survey of CAM use in an eastern region of Germany also found that a significant proportion of CAM users were current smokers (28.6%) [37] Published English-language reports of population-based surveys of CAM use in non-U.S. population are sparse. Most published reports focus on specific clinical populations, e.g. outpatients to a health center, cancer survivors. Some clinical population studies have reported significant rates of tobacco use among CAM users. A Swedish health centre-based survey of 1442 patients found that among users of manual manipulative CAM therapies, 14.7% were current smokers, and 18.8% were current snuff takers. Of those using manipulative CAM therapies and herbs, 25.6% were current smokers, and 37.5% were current snuff takers [38]. In the U.S., as in some other countries, some populations with higher rates of CAM use are also at higher risk for tobacco use. These populations include: the uninsured/underinsured [4,7,39]; some low-income and rural populations [40-42]; some ethnic/racial minority and new immigrant groups and persons living with specific conditions such as HIV/AIDS [43,44], mental illness [45,46] and cancer [47-49].

Despite their increasingly important role in healthcare, and potential to promote tobacco cessation, CAM practitioners have largely been overlooked in the public health tobacco control agenda. Further, because of the different professional background and training, clinical practice models, scope of practice and practice patterns that clearly distinguish CAM practitioners from conventional biomedical practitioners, existing tobacco cessation training programs designed for conventional practitioners may not be well-suited for CAM practitioners.

To ensure that the CAMR intervention had context validity for the three CAM disciplines engaged in this study, we used an iterative and community based participatory research (CBPR) approach to develop an intervention protocol integrating conventional tobacco cessation interventions recommended by the PHS Guideline into real world CAM practice. The CAMR intervention builds on an existing program of research [25,50-53] that developed the Helpers Program (“Helpers”), the foundational curriculum for the CAMR intervention. Helpers is a community-based brief intervention (BI) training program that teaches lay community members how to offer a structured, four-step “helping conversation” to tobacco users. The helping conversation uses active listening skills and motivational communication strategies to encourage quitting tobacco (i.e. permanently stopping/giving up tobacco) without confrontation, or “nagging” [53]. One of the inspirations for CAMR came from local CAM practitioners (chiropractor, acupuncturist, and massage therapist) who had participated in a prior NCI-sponsored study of community-based tobacco cessation BI training for the lay public (Project Reach) [25]. These CAM practitioners recognized the value of such training for their own practices, and at the end of the study approached the research team with the recommendation and request that cessation training, tailored to the needs of CAM practitioners, be offered to their disciplines. The purpose of this paper is to describe the iterative CBPR process we used to develop the CAMR intervention protocol and the resulting intervention that included both a patient/client centered tobacco cessation BI training and a practice system intervention tailored for CAM practitioners. We note that the CAM disciplines participating in the CAMR study customarily use different terms to refer to persons seeking their care. Chiropractors and acupuncturists usually refer to “patients”, whereas massage therapists usually say “clients”. For simplicity, we will use “patients” throughout this paper.

## Methods

The CAMR intervention development was designed as an iterative process in which the outcomes from each step were used to inform and shape the next step. The six steps for each type of CAM practitioner were: 1) exploratory interviews with key informants; 2) survey of local CAM practitioner community members; 3) test demonstration of existing curriculum with CAM practitioners; 4) adapting and tailoring existing curriculum; 5) external review of adaptations; 6) delivery of tailored curriculum to CAM practitioners with follow-up curriculum evaluation by trainees. Methods and process outcomes for each developmental step are presented below. The resulting curriculum and intervention then moved into the main research study protocol, described elsewhere. The University of Arizona Human Subjects Protection Program approved the study (Protocol No. 0900000349R002). Informed consent was obtained from each participant involved in the study.

### Step 1 – Exploratory interviews with key informants

#### Methods

The purpose of the key informant interviews was twofold: to gain a better understanding of potential barriers and facilitators that practitioners may have to conducting helping conversations so that these factors could be directly addressed within the intervention, and to inform development of the community survey of local CAM practitioners for Step 2. Initial key informants (chiropractic, acupuncture and massage therapy) were identified through project investigators’ existing relationships with CAM practitioners in the local Tucson community. These informants referred investigators to additional practitioners for invitation to participate in individual, semi-structured interviews. A total of eleven practitioners participated (3 chiropractors, 3 acupuncturists, and 5 massage therapists). The three chiropractors were in private solo practice. One chiropractor was primarily providing *locum tenens* coverage for other chiropractors in the community. One chiropractor was also a licensed acupuncturist. He used acupuncture and nutrition advice within his practice, but self-identified as a chiropractor. One chiropractor had originally trained and practiced as a massage therapist before going to chiropractic school, but self-identified as a chiropractor. He predominately used chiropractic manipulation treatments and rarely used massage therapy in his practice. All three acupuncturists were trained in acupuncture and Traditional Chinese Medicine (TCM). Two were in private solo practice; one was also part-time faculty at a local acupuncture/TCM school. The third acupuncturist was president and faculty of an acupuncture/TCM School. He had an active practice within the school and was also faculty for the University of Arizona College of Medicine’s Program in Integrative Medicine. The five massage therapists represented a range of practice contexts and used a range of massage/bodywork treatments (Swedish, deep tissue, shiatsu, ergonomic evaluation). Three massage therapists were working in relatively large group practices (>5 practitioners); one was a practice owner/practitioner, one as an independent contractor and one as an employee (who also had a mobile private solo practice, delivering treatments in patients’ homes). The other two massage therapists were in private solo practice; one also had a mobile practice. Three massage therapists were current or former faculty at local massage therapy schools.

These practitioners were chosen because they were: 1) experienced practitioners in their CAM discipline; 2) in active clinical practice at least half-time; 3) known and respected by their professional peers in the local community; and 4) had experience with either CAM professional education, clinical research, and/or elected leadership in one of their discipline’s professional organizations. Only one practitioner (chiropractor) had prior special interest in tobacco control and had sought out conventional tobacco cessation training. Three practitioners identified members of their office staff as having key roles in practice flow or patient education; these staff members were also interviewed (n = 4). Office staff roles were receptionist (n = 2) and treatment assistant/patient educator (n = 2). Several of the key informant practitioners continued their engagement with the project by becoming members of CAMR’s Local Advisory Panel. This panel provided on-going local practitioner input and pilot testing of the subsequent project intervention and evaluation. The panel consisted of 2–4 practitioners from each of the three CAM disciplines involved in the study.

Investigators conducted open-ended, semi-structured interviews with key informants, usually at the practitioner’s office. The interview guide addressed the following domains: practitioners’ attitudes, knowledge, prior training, experience and practice behaviors regarding tobacco dependence and cessation. Also queried were: attitudes, practices and experience with counseling patients/clients on other lifestyle-related issues; patient and practice characteristics; and practice flow and logistical issues important to the intervention model.

We probed particular specific areas of possible practitioner concern: e.g. how they felt about talking to patients about tobacco when they come in with a specific presenting problem; whether in their experience patients see questions about tobacco as odd or intrusive, or as a routine part of a holistic practitioner’s intake evaluation; whether practitioners were concerned about losing patients if practitioners became more proactive in tobacco cessation counseling, and if practitioners saw their discipline as having a role in public health in general and tobacco control specifically.

Interviews were audiotaped and transcribed for later thematic analysis. All transcribed interviews were coded manually for a basic set of themes identified from an initial reading of all interviews. Themes reflected both key investigator interest areas as well as issues that emerged from unprompted discourse. Attention was paid to the multi-vocality of informants who expressed divergent opinions at different points of the same interview indicating shifts in context.

### Step 2 – Survey of local CAM practitioner community

#### Methods

The survey of local CAM practitioners was designed to query respondents on domains that might impact acceptability of the CAM Reach intervention and that were found to be salient in the key informant interviews in Step 1. Findings of this step were intended to inform questions that would be asked of participants in the demonstration training in Step 3 so that curriculum content could be tailored for each CAM discipline. The survey was pilot tested with key informants from Step 1, augmented by approximately eight additional local practitioners recruited through Step 1 key informants and research team’ personal contacts. A letter introducing the practitioner survey was mailed to all practitioners with an address in the Tucson, Arizona metro area. Address lists were obtained from the Arizona state licensing boards for chiropractors, acupuncturists and massage therapists (N = 1560; chiropractors (N = 187), acupuncturists (N = 126), massage therapists (N = 1247). An introductory email was also sent out through an alumni email list for two local massage therapy schools. The one-page practitioner survey was mailed out two weeks after the introductory communications. The survey queried: years in practice, which CAM disciplines are practiced, prior tobacco cessation training, screening for tobacco use, cessation advice, interest in receiving cessation training, and interest in research participation. No incentives were offered for survey completion. Survey non-responders received one follow-up telephone call, asking practitioners to complete the survey.

We described the survey data using means and standard deviations, tabulating for sub-populations using Stata 11 [54].

### Step 3 – Demonstration of existing tobacco cessation curriculum to CAM practitioners

#### Methods

In order to evaluate the extent to which an existing training might be useful as a foundational curriculum for CAM Reach and to identify key areas for tailoring, we demonstrated the Helpers Program (Helpers) an existing tobacco cessation training developed in our prior research [53], for a group of local CAM practitioners in Step 3, including some of the key informants from Step 1. The aims of Step 3 were to assess overall acceptability of tobacco cessation training content based on the PHS Guideline, and gather practitioner feedback on the type and extent of revisions needed to tailor the training for each CAM discipline. Helpers is a multimedia, interactive training that emphasizes a tobacco-user centered, non-confrontational and motivational approach to tobacco cessation [53]. More specifically, this curriculum addresses: tobacco addiction (to build empathy toward tobacco users struggling to quit; communication skills (e.g. active listening) that specifically guide Helpers away from confrontation or nagging; assessment of readiness to quit (to reduce the inclination to push tobacco users with low readiness to quit); and referral skills to connect tobacco users with established cessation services (e.g. telephone-based tobacco cessation counseling services, or “quit lines”) along with basic information regarding cessation aids recommended in the PHS Guideline [19].

Helpers incorporates key components of the PHS Guideline recommended 5As [19], but transforms the traditional application of the 5As into a less proscriptive approach that is more tobacco-user centered, and focuses on encouraging tobacco user behavior change that is aligned with the tobacco user’s current willingness/readiness to take action toward giving up tobacco. This is because the 5As’ proscriptive approach to tobacco cessation was originally developed to guide allopathic physicians in helping patients quit tobacco. The 5As presumes a provider-patient relationship context and places much emphasis on advising a tobacco user to quit, regardless of their readiness to do so. In contrast, the helping conversation is a 4-step approach that does not presume a particular health care context, is motivational and more tobacco-user centered, and focuses on encouraging behavior change that is in the direction of quitting, yet aligned with the tobacco user’s current willingness/readiness to take action toward quitting.

To garner feedback about Helpers training for CAM practitioners, the research team held a one-day Helpers training demonstration and debrief/critique session with seven local CAM practitioners (two chiropractors, three acupuncturists, and two massage therapists) followed by a focus group-type feedback/debriefing session. Practitioners were recruited from key informants, key informant referral and our research team’s personal contacts with local CAM practitioners. The number of practitioners recruited was based on practical considerations of having representation of each of the CAM disciplines in the study, and a range of practice styles to likely achieve saturation of feedback themes. Practice styles of participants were very similar to those of key informants Step 1. Participants were asked to participate as practitioners-trainees, but also to take notes on feedback forms for the afternoon debrief/critique session. Forms were structured to help elicit specific and detailed feedback on each section of the training. One CAM practice staff member (receptionist) also participated. After the training demonstration, participants were also shown materials from a 5A’s tobacco cessation training developed for chiropractors (patient handouts, practitioner guides, and display posters) [33]. Participants gave feedback about the utility of these materials in their practices and agreed to pilot test the CAM Reach system intervention components in their practices (display materials, chart reminders, practitioner guides, patient handouts). Investigators analyzed their notes from the demonstration session (direct observations and practitioner verbal comments) and practitioners’ written feedback for overarching and convergent themes of (e.g. acceptability of training content) as well as specific critiques and suggestions for revision. Approximately 1–2 weeks after the demonstration training, project staff went to each practitioner’s office to conduct observations of the practice flow and practice environment. At this visit staff also pilot tested the feasibility and acceptability of employing an in-office “practice patient” (standardized patient) as a final learning activity/readiness assessment to conclude the CAMR training. Feasibility was assessed by conducting the practice patient role-play in each practitioner’s practice location with research staff noting the ease (or difficulty) of completing the practice patient role-play. Immediately after concluding the role-play activity, staff solicited practitioner acceptability feedback about the practice patient experience, e.g. practitioner comfort/discomfort with activity, perceived value as a learning experience, recommendations for inclusion/exclusion as a learning activity in the final CAMR intervention, and suggestions for improvement. Approximately 2 months later, practitioners participated in a follow-up focus group discussion and feedback about experiences using the new tobacco intervention skills and practice support /system intervention materials. Findings from this step were used in the adaptation and revision of the existing curriculum in Step 4.

### Step 4 – Adaptation and revision of existing curriculum

#### Methods

The aim of Step 4 was to adapt the existing Helpers curriculum for context validity in each of the three types of CAM practices included in this study, in preparation for external subject matter expert review in Step 5. Practitioner feedback from Step 4 was analyzed for consistent themes and convergent recommendations for change. The Helpers training curriculum was deconstructed into major and minor topical areas to facilitate adaptations of existing content areas, addition of new content, and rearranging the order of topic presentation. The basic organization of the training modules corresponded to each of the four steps of the helping conversation (Awareness, Understanding Helping, Relating) was retained.

### Step 5 – External subject matter expert review

#### Methods

A ten member national advisory panel reviewed the adapted curriculum with the purpose of providing feedback and advice for further necessary revisions. Advisory panel members were selected for their nationally/internationally recognized subject matter expertise in: education and/or research in one of the three CAM disciplines included in the study (two chiropractors, two acupuncture/Oriental medicine practitioners, and two massage therapists); two integrative medicine (two practitioners); and tobacco cessation research and policy (two experts). Advisors provided structured feedback on: 1) adequacy and appropriateness of desired learner competencies, 2) overall instructional approach; 3) learning goals, objectives and instructional activities; and 4) completeness of content.

All advisors were sent a curriculum review package containing: 1) reviewer instructions, 2) draft of core competencies for learners’ training goals, 3) detailed outline of the adapted training curriculum and descriptions of learning activities, 4) reviewer feedback forms which queried: the CAMR intervention’s validity/suitability for the CAM practice context; training content appropriateness and completeness; instructional design, length and format acceptability; and opinions regarding dissemination potential. The curriculum development team compiled and analyzed national advisor feedback for over-arching and recurrent themes as well as convergence of specific critiques and recommendations. National advisors then participated in a follow-up conference call to discuss, clarify and expound upon the panel’s collective feedback. The findings of this stage were integrated into the curriculum in preparation for final pilot testing in Step 6.

### Step 6 – Pilot test of revised curriculum

#### Methods

The new CAM Reach (CAMR) training (1-day training workshop and the follow-up in-office standardized patient exercise) was tested in a pilot of the full training with a second (new) group of CAM practitioners (N = 8), along with investigators (N = 2), and project staff (N = 4) to confirm integration of results from prior development steps and fine-tune presentation timing and use of multimedia. Practitioners were recruited through key informants, key informant referrals, and the research teams’ personal contacts with local practitioners. The number of practitioners recruited was based on practical considerations of having representation of each of the CAM disciplines in the study, and a range of practice styles thought to likely achieve saturation of feedback themes. Practice styles of participants were similar to those of key informants Step 1. The workshop also distributed a number of practice support printed materials described in more detail below (e.g. display posters, informational brochures, stickers flag charts of tobacco users). CAM practitioner attendees at this pilot training had not participated in the demonstration training described above nor had prior exposure to the training content. The data collection process was the same as in Step 3. Practitioners were asked to participate as trainees/reviewers, while taking written notes on forms specific to each section of the training to provide feedback in the debriefing/critique session held immediately after completion of the 1-day workshop. The debriefing session was audio-recorded to provide back up for investigator and project staff notes taken during the session. Research team notes and practitioners’ written feedback were analyzed for consistent themes, convergent critiques and recommendations, which informed the revisions leading to the final CAMR training workshop component of the CAMR intervention protocol.

## Results

### Step 1 results– Exploratory interviews with key informants

Step 1 aimed to better understand CAM practitioners’ potential barriers and facilitators to conducting helping conversations. Major themes from the interviews are summarized in Table 1.

**Table 1:** **Major themes from key informant interviews**

**Interest in CAMR Study**	Thought tobacco cessation was relevant and important to practice; CAMR and participation in tobacco cessation viewed as a public health service
**Experience w/ Tobacco use (TU) Conversations**	TU conversations not typically initiated by practitioners unless requested by client/patient; TU not uniformly assessed among new clients/patients; practitioners felt most comfortable with initiating conversations about TU with established clients/patients
**Barriers to TU Conversations**	Patient might perceive TU conversations as intrusive - potential client alienation or confrontation; being perceived as giving a “sales pitch”; time constraints; cost effectiveness of TU conversations; scope of practice concerns (massage therapists); potential for patient to be dissatisfied and leave the practice
**Training Content Desired**	Tobacco use effects on health and the healing process, link between tobacco use and common presenting problems; TU conversation starters; biomedical and psychological perspectives of tobacco addiction; TU cessation referral resources
**Tone Desired**	Encouraging, supportive, focused on listening and referral
**Environmental/System Change**	Intake appointments typically long, allow for lifestyle conversations; return client flow allows for follow up conversations; intake forms could be modified to include TU questions; posters and handouts welcomed in practice
**Research/Training logistics**	No-cost training and CEUs extremely desirable; practice patient protocol acceptable and positively regarded

Practitioners uniformly felt that tobacco use was detrimental to patient’s health and that cessation training was relevant to their practices. Three practitioners also viewed engaging in tobacco cessation as a public service or public health role for their CAM discipline.

*“And I think there’s a lot of chiropractors there, and they see a lot of patients, and this [tobacco cessation] would be one way—chiropractic is supposed to be about creating a healthier body, and therefore, I think chiropractors are perfect for this [promoting tobacco cessation]. And I think the profession as a whole, if some chiropractors got involved, the American Chiropractic Association, would throw their full support to chiropractors doing something like this, because I would think it would only help chiropractors to be seen as doing more of a public service. [RS, chiropractor]*

Two frequently cited barriers to addressing tobacco use with patients/clients were similar to those encountered among conventional practitioners, i.e. time constraints [55] and lack of training.

*“Some [chiropractors] are high volume and won’t take much time, but others will.” [KS, chiropractor, talking about barriers to talking to patients about tobacco cessation]*

*“I hadn’t really thought about why is it I’m not seeing smoking cessation and like I said, I never felt that successful at it, initially, and then, so people have called me and I’ve started to deflect; ‘Why don’t you see someone else who specializes in this?’” [LM, acupuncturist, talking about why she does not routinely address tobacco use in her practice]*

*“I bet there’s a lot of new information that I’m not aware of, the whole neuro-transmitter thing, I bet there’s a lot of great stuff that I should know. And it would probably prompt me to think about how I use acupuncture and how I might go, OK, if I can understand it in this neurological way, this modern way, how would I bring my acupuncture ideas to bear on that, that would interest me a lot. ‘Cause I think that that piece about any addictive substance is so interesting.” [LM, acupuncturist, talking about her interest in receiving cessation training]*

*“ I probably do not bring it up, um, and I let the client bring it up first, then maybe would go into, the physical effects of that and how its affecting the condition that maybe they’re complaining about, but I think I would also need more information about that too, I don’t think—My study at massage school, I actually did research on using massage with alcoholism, but not with tobacco.” [CR, Massage therapist, talking about barriers to talking about tobacco use with clients]*

Barriers that differed from those commonly cited by conventional practitioners were: perceived intrusiveness or potential patient/client social discomfort or alienation– i.e. social risk [34] and concerns over whether addressing tobacco use fit within their scope of practice.

*“… when [they are] on the massage table, because they’re naked, and there's a sheet over and they're laying down and I’m standing up and I'm clothed, I've tried to avoid anything that would increase that power differential or increase, maybe, a shame level.” [GA, Massage Therapist]*

*“… so we really have to be extremely careful when we’re making suggestions. There is a away that you can make suggestions based on your own personal experience, or somebody else’s experience that sounds similar to theirs…just passing on that information, you are not prescribing or diagnosing and so we do that sometimes, but we really have to be careful with that.” [CR, massage therapist, talking about discussing tobacco use with client and scope of practice]*

Practitioners expressed more hesitancy to bring up tobacco use with new patients, preferring to defer addressing tobacco use until later in the relationship. Two practitioners were concerned that raising the issue of tobacco could potentially to be perceived by patient as a “sales pitch” for additional CAM services.

*“I think it’s easier done [bringing up smoking] when you have a patient relationship, which is built over the years, it’s much easier to deal with it. You know, if you bring it up to a new patient on a second visit, then it’s sort of you don’t have the trust bond that you do with your older patients.” [RS, chiropractor]*

*“I could see myself doing it in the clinic maybe after a session, if the conversation had come up, if we were talking about—if they were asking me questions about it, then definitely. Or if I had to approach it with them, I would do it very carefully, in a roundabout way most likely, and then try to have them bring themselves into it. …. I’d want to make sure that they obviously are interested in quitting because it really needs to be them. That’s why I usually let them come to me.” [DD, Massage therapist, talking about speaking to clients about quitting tobacco]*

Practitioners conveyed frustration with the difficulty of motivating patient behavior change related to lifestyle issues, the associated paucity of sustained behavior change, and patients’ frequent expectations of a “quick and easy” fix – echoing sentiments often expressed by conventional medical practitioners.

*“… one woman I’ve seen off and on for many years, I tried to help her quit smoking with acupuncture and it didn’t work. Now she’s finally quitting. She’s tried and tried and tried. Finally, she’s quitting with that drug Chantix.” [LM, Acupuncturist, talking about difficulty of sustained behavior change]*

*“But what I felt like was, some of the people I worked with who were smoking cigarettes, they were really hoping it [acupuncture] would be magic, and that they wouldn’t have to do any of the emotional work of really looking at the addiction.” [LM, Acupuncturist]*

*“But I do have cases where people are not ready. I think people believe that this [acupuncture] can make them quit. I said, nothing under the sun can make you quit, when you are ready to quit, then you can come to me, and I’ll help you quit. But don’t think that these cigarettes can erased your memory; that you’ve never been smoking before, that you never knew what smoking is all about.” [SL, Acupuncturist]*

Informants were also asked whether they thought that a learning/assessment activity that featured an in-office “practice patient” (standardized patient) as a way to evaluate and clinical skills and receive feedback would be useful and acceptable. Participants thought this an interesting idea, likely to be clinically useful and well accepted. Practitioners reported two factors that would encourage their participation in tobacco cessation training: being free of charge and practitioners would receipt continuing education credits for training participation.

Data from Step 1 led us to develop a sensitive and context-driven approach to how and when to approach different patients about tobacco use. It also led us to document that participating practitioners found their patients to be receptive to tobacco conversations.

### Step 2 results - Survey of local CAM practitioner community

Step 2 aimed to gather information from the local CAM practitioner community on domains potentially effecting acceptability of the CAM Reach intervention (based on results of Step 1). Overall survey response rate was 23% (n = 356), with differences in response rate by discipline: chiropractors, 30% (n = 56); acupuncturists 50% (n = 63); massage therapists, 19% (n = 237). Overall, nearly two thirds (64.6%) of those responding reported no previous cessation training. Prior cessation training was most common among acupuncturists and least common among massage therapists. Practitioners reported infrequently advising patients/clients to quit tobacco. Approximately two-thirds of practitioners responding were interested in receiving cessation training. See Table 2.

**Table 2:** **Prior tobacco cessation training, interest in training by practitioner type**

	**Overall**	**Acs**	**DCs**	**MTs**
	**(n = 356)**	**(n = 63)**	**(n = 56)**	**(n = 237)**
	**%**	**%**	**%**	**%**
Prior Cessation Training				
None	64.6	8.5	66.1	78.9
In professional school	19.9	72.9	17.9	6.6
Cont. Education	11.1	35.6	16.1	3.5
Learned on own	17.3	37.3	12.5	13.2
Interest in Cessation Training				
Yes	66.4	62.3	66.7	67.3
No	10.3	13.2	4.4	10.8
Unsure	23.5	24.5	28.9	22.0

Acs = acupuncturists; DCs = doctors of chiropractic; MTs = massage therapists.

### Step 3 results - Demonstration of existing tobacco cessation curriculum

This step aimed was to evaluate an existing training (Helpers) as a foundational curriculum for CAM Reach and to identify key areas for tailoring. Practitioners reacted positively to the Helpers overall training content and instructional approach, including the patient-centered, motivational focus of the structured helping conversation. They demonstrated keen interest in the pathophysiology of tobacco’s health effects as well as the conventional/PHS guideline-based therapies, particularly cessation medications, wanting to know more so that they would feel comfortable responding to patients’ questions. In the training debriefing, practitioners asked numerous questions and recommended expansion of these two content areas of the training. Despite prompting by investigators, practitioners showed much less interest in hearing more about CAM therapies for cessation. Practitioners wanted inclusion of new and different training tools and patient handouts (e.g. handouts addressing the link between tobacco use and common presenting problems of patients; a detailed handout about medications that could be provided to interested patients; and a quick reference of benefits of quitting) and recommended additional skill-building activities in the instructional design. They also made suggestions for types of video role-plays (e.g. depicting practitioner interactions with patients who were more resistant to talking about their tobacco use, as well as receptive patients) and practitioner interview clips for the multi-media aspects of the training. Despite the differences in professional backgrounds and scope of practice among the three CAM disciplines, there were no recommendations for discipline-specific tailoring other than inclusion of interview clips from the same CAM discipline as the practitioner audience. Practitioners also saw value in keeping the interview clips from different CAM disciplines and did not recommend limiting clips to practitioners from the same discipline as the audience. Practitioners uniformly viewed the in-office “practice patient” (standardized patient) learning activity as a positive, informative experience and supported its inclusion in the final study intervention protocol.

### Step 4 results – Adaptation and revision of existing curriculum

This step aimed to adapt the existing Helpers curriculum for context validity for each of the three CAM disciplines included in this study. A key conceptual adaptation of the curriculum was to emphasize the role of the relationship between practitioners and patients/clients. The CAM Reach training was framed as based on three fundamental principles: 1) tobacco cessation is a process, not an event; 2) practitioners can offer helping conversations to a tobacco user at any stage in the process of quitting; 3) helping conversations are part of a supportive, healing relationship.

Specific content was added to address second-hand smoke, and third-hand smoke exposure, and to provide minor expansion of CAM therapies content to address current research about CAM therapies for cessation. Content was added on screening for second-hand smoke exposure in non-tobacco users. A referral resource for patients who were interested in helping the sources of their second-hand smoke exposure – usually a friend or family member - to give up tobacco was also added. This resource is the Helpers Program on-line training, described above [53]. Finally, learning activities were expanded and arranged so that participants would have progressive practice with helping conversation skills over the course of the training, with a summative skills practice role-play at the end of the training. A standardized “practice patient” experience was added as a summative learning activity/skills evaluation to be administered in the practitioner’s office approximately two weeks after the training workshop. The workshop content was reconfigured into an introduction and four modules (Table 3). The total training length was expanded to eight contact hours (7 hour workshop plus 1 hour in-office standardized patient). The final workshop was accepted for eight hours of continuing education units by the Arizona licensing boards for chiropractic, acupuncture and massage therapy.

**Table 3:** **CAM reach training curriculum modules**

**Training module**	**Content**
Introduction	Overall knowledge and skills goals for the training, three guiding principles of Reach training, four steps of a Helping conversation, video example of helping conversation between practitioner and patient.
Module 1 - Awareness	Scope of the tobacco problem, tobacco’s effects on health and healing, importance of linking effects of tobacco use to patient’s health concerns, practice systems to identify tobacco use, harm from second hand and third hand smoke exposure, the CAM practitioner’s role in helping, context of helping, getting the helping conversation started, skills practice role play
Module 2 - Understanding	Tobacco products and their harmful constituents, aspects of tobacco addiction (biological, psychological, social), active listening and communication skills (open-ended questions, reframing, body language), motivators and barriers to quitting (i.e. giving up tobacco), assessing readiness to quit, skills practice role play
Module 3 - Helping	PHS guideline, types of cessation behavioral support services, cessation medications, referral skills, CAM approaches for tobacco cessation, motivational strategies (i.e. motivating and clarifying questions, eliciting ‘change talk’, ‘rolling with resistance’, emphasizing benefits of quitting, negotiating action), importance of continuing to offer helping conversations – even with patients not ready to quit, components of a simple quit plan, skills practice role play
Module 4 - Relating	Finishing the helping conversation on a positive note, setting the stage/leaving door open to have future helping conversations, tips and strategies for following up, two final skills practice role play
Closure	Distribution of printed practice support materials, discussion of how to use/implement printed materials to engage patients and promote practitioner’s willingness to help tobacco users quit, explanation of practice patient (standardized patient) office visit

### Step 5 results - External subject matter expert review

The purpose was to gather feedback and advice for further necessary revisions from nationally/internationally recognized experts in the three CAM disciplines, tobacco cessation, and integrative medicine. Congruent with results from Step 3, national advisors also supported the interprofessional education approach, recommending only a minor amount of tailoring for each practitioner type. There was also strong support for the conceptual shift toward a relationship-centered intervention approach with an instructional design and activities emphasizing progressive skills building. National advisors also provided substantive contextual input on specific issues including: typical content/training received in typical CAM school curricula, professional scopes of practice, integration of conventional therapies, and potential practitioner role in discussing/providing information on cessation medications. National advisor feedback and contextual information informed additional tailoring of curriculum content, patient handouts, and instructional design for the unique needs of chiropractors, acupuncturists and massage therapists. Advisors also commented on the dissemination potential of the proposed CAMR intervention and recommended exploration of online training possibilities as well as integration of CAMR tobacco cessation training into CAM primary professional education settings.

### Step 6 results – Pilot test of revised curriculum

Step 6 aimed to confirm integration of results from prior development steps and identify last revisions needed to produce the final CAMR training intervention. Participant feedback confirmed that national and local advisor recommendations had been effectively incorporated and also recommended the elimination of one learning activity that was felt to be overly technical and not helpful to explain or reinforce content. In particular, CAM practitioners in attendance were very positive about the new content on pathophysiology of tobacco health effects and cessation medications. Practitioners commented that although they felt any recommendation to use medications was outside of their scope of practice, they noted that patients frequently ask them about medications (both over-the-counter and prescription). Practitioners found the medication information interesting and useful in that they were now more comfortable with offering the CAMR patient handouts about medications and/or directing their patients to physicians, pharmacists or “quit lines” (free telephone-based stop smoking counseling services that are widely available in all U.S. states) for more information and assistance with cessation medications. Practitioners liked that the CAMR training resulted in new knowledge and skills that were immediately applicable in their practices. Other feedback included recommendations for minor re-ordering of slides, video role-plays, and practitioner testimonials for better instructional flow. As in Step 3, the in-office standardized patient exercise was uniformly viewed as a positive and very helpful learning experience.

### Final CAMR intervention protocol

The final CAMR intervention protocol and content is outlined in Table 4. Broadly, the protocol called for both practitioner education and system change components that create a welcoming and information rich environment for patients. For example, there were seven different display posters, stickers with tobacco screening questions for intake forms, chart stickers (to signify tobacco users). The display posters depicted a variety of people with text encouraging patients/clients to ask their practitioner about quitting tobacco or second hand smoke, e.g. “Ask your [practitioner type] about quitting tobacco”. One poster’s text addressed pain: “Did you know that smoking can increase your pain? Ask us for help to quit”.

**Table 4:** **Final CAM reach intervention protocol**

**Intervention component**	**Description**
CAMR training workshop	7 hour, in-person continuing education workshop (7 CEUs)
Practice patient/system change visit	1 hour in-office visit to conduct practice patient assessment and help implement office system changes (1 CEU)
Patient education materials	10 brochures:
	Tobacco and Your Body: Surprising things that you may not know; Secondhand and Thirdhand Smoke: Surprising things that you need to know; Thinking of Quitting Tobacco? We Can Help; Medications that Help with Quitting Tobacco; The Personal Quit Plan; Simple Quit Plan; Quit Line brochure; Helpers Brochure (for those wishing to help others quit tobacco); Roadmap for Quitting Tobacco; Benefits of Quitting Timeline
Practice support materials	Display posters, intake form stickers, chart stickers, brochure holders
	7 different display posters, stickers with tobacco screening questions for intake forms, chart stickers (to signify tobacco users). Display posters depicted a variety of people with text encouraging patients/clients to ask their practitioner about quitting tobacco or second hand smoke, e.g. “Ask your [practitioner type] about quitting tobacco”, and “Got pain? Did you know that quitting tobacco can help? Ask us how”.

## Discussion

Researchers conducting CAM research have consistently faced methodological critiques of interventions that lack context validity within real world CAM clinical practice. The CAMR intervention protocol development process addressed context validity from both the perspective of CAM practitioners as well as conventional biomedicine. Incorporation of the latest thinking in tobacco cessation from conventional research as well as formative research with CAM practitioners was essential to the formulation of the three guiding principles of the CAMR intervention: 1) tobacco cessation is a process, 2) practitioners can offer helping conversations to a tobacco user at any stage in the process; 3) helping conversations are part of a supportive, healing relationship.

By attending to context validity, the CAMR intervention was able to bridge a gap between the proscriptive 5 A’s approach the PHS Guideline recommends for conventional biomedical practitioners (i.e. ask about tobacco use at every visit and advise the user to quit) and the relatively greater hesitancy of CAM practitioners to bring up tobacco use with new patients. The final CAMR intervention emphasizes a motivational, relationship-centered approach to the helping conversation, in which the four steps of a helping conversation (Awareness, Understanding, Helping, Relating) are sequenced to help the practitioner address tobacco use, while attending to the relationship. For example, the Awareness step prompts the practitioner to identify links between the patient’s tobacco use and *their reasons* for seeking treatment and to offer the patient information, thus laying groundwork for addressing tobacco use now, or at a future visit. The Understanding step helps the practitioner to attend to the relationship by asking about the *patient’s* reasons for wanting to quit tobacco and *their* readiness to quit tobacco before offering Helping (e.g. advice, information, motivational strategies) that is in alignment with the patient’s acceptance and readiness to take action. Finally, Relating emphasizes the practitioner’s role in attending to the relationship by seeking permission to follow-up and providing ongoing support for behavior change.

The iterative development process also yielded some interesting outcomes. First, the participating practitioners expressed much more interest in having more information about biomedical models of the mechanisms of tobacco’s health effects and also cessation medications, than additional information about CAM therapies specifically for tobacco cessation. Discussion with national advisors, indicated that practitioners were likely to be already familiar with therapies from their own system of treatment.

Second, the development process did not identify a need to extensively tailor the CAMR intervention for each CAM discipline. Rather, participating practitioners’ and advisors’ comments confirmed earlier formative research results about the course content (information and skills training) as having relevance and clinical application across different CAM disciplines (e.g. body-system specific health consequences of tobacco use, communication skills). Notably, practitioners spontaneously identified other health behaviors that might be addressed using the same communication skill set. Practitioners also pointed to the potential for interprofessional education – the opportunity for practitioners from different CAM disciplines to learn from one another *vis a vis* such conduits as videos modeling how practitioners from another CAM discipline approached patients about tobacco their use. An interprofessional approach to training is particularly relevant for those who practice with CAM practitioners from other disciplines – a common scenario [56]. A third interesting outcome were the similarities between the frustrations expressed by CAM practitioners and conventional practitioners over the challenges of motivating patients/clients to make and sustain healthy behavior changes.

A limitation of the study is that the participating CAM practitioners self-selected to be in a research study on tobacco cessation, and thus may not be fully representative of the general population of CAM practitioners. There were a limited number of CAM practitioners participating in the development steps (other than the mail survey). These practitioners also self-selected to participate in an intervention development process, so their results may not be generalizable. Another limitation is the low response rate of chiropractors and massage therapists in Step 2. It is possible that the high proportion of respondents with no previous cessation training, and an interest in receiving cessation training is over estimated. Such practitioners may have been more likely to answer a survey about tobacco cessation training and may not reflect the actual need or demand for cessation training among the general population of CAM practitioners.

Acupuncturists’ higher response rate to the community CAM practitioner survey may be a reflection of more acupuncturists reporting having had prior training in tobacco cessation, either in their primary professional training or as continuing education. This may indicate greater interest and/or familiarity with the topic of tobacco cessation and a higher likelihood of responding to a survey about tobacco cessation training. Of the three CAM disciplines participating in our study, only acupuncturists have specific treatments within their core practices that are for treatment of drug withdrawal. Our national advisors indicated that there is a well-known acupuncture protocol for treating drug withdrawal that can be applied to nicotine withdrawal, and that this protocol is typically taught in acupuncture school. The present study was conducted in the U.S. Primary professional training, scope of practice, and government or industry regulation of CAM practitioners in other countries may be different. Accordingly, care must be taken in any transferability and generalizability of study findings and the resulting CAMR intervention protocol to CAM practitioners in other countries.

In conclusion, the CAMR intervention protocol, with its focus on patient-centered care and the role of the patient-practitioner relationship, has potential to serve as a common touchstone that has context validity yet could generalize across three vastly different CAM disciplines and their varied practice contexts – and connect practitioners in a way conducive to interprofessional education and practice. More importantly, can the CAMR intervention change CAM Practitioner clinical behavior in real-world practice settings? This question is the focus of the practice-based CAMR study and must be answered be answered before wider adoption of the CAMR intervention protocol. A related research question follows: Could the same common focus on patient-centered care and the patient –practitioner relationship also help bring together both conventional and CAM Practitioners in collaborative efforts to help patients give up tobacco use? With the growing interest by conventional health practitioners and the public in integrative medicine, and CAM practitioners’ growing interest in ways to enhance their contributions to public health education and promotion, this question also deserves further research.

Shared frustrations over motivating patients to make and sustain healthy behavior change are common among practitioners of all types, providing a departure point for productive dialogue and exchange of experiences. A common desire for more effective ways to promote healthy behavior change provides an opportunity for collaboration in what we have elsewhere described as a community of cessation practice [57]. This desire can serve as the basis for cessation training in a shared repertoire of behavior change strategies and tools, e.g. helping conversations, active listening skills, and motivational communication strategies that could help bring CAM and conventional practitioners together toward a common goal of reducing tobacco use [57].

## Conclusions

CAM practitioners are well suited to delivering tobacco cessation brief interventions to their patients and clients - they have access to tobacco users, motive to take action (desire to promote health, healing and wellness) and opportunity to intervene (patient/client contact time). An inclusive and iterative process to develop the CAMR training curriculum and practice intervention, with much formative research, resulted in an intervention protocol that has context validity for CAM practitioners in that it: 1) is patient-centered and emphasizes the practitioner’s role in a healing relationship; 2) is practitioner-friendly in that it is responsive to the different contexts of CAM practitioner practices and their patient relationships; 3) integrates relevant best practices from U.S. PHS Clinical Practice guideline on treatment of Tobacco Dependence; and 4) is suited to the differing contexts of healing philosophy, scope of practice and practice patterns found among CAM practitioners. The CAMR practice-based mixed-methods research study currently underway in a larger sample of CAM Practitioners (N = 99) will evaluate the effectiveness of this intervention protocol in changing CAM practitioners’ clinical practice behavior.

## Abbreviations

Acs: Acupuncturists
CAM: Complementary and alternative medicine
CAM: Practitioners (chiropractors, acupuncturists, massage therapists)
CAMR: CAM reach project
DCs: Doctors of chiropractic
MTs: Massage therapists
PbCAM: Provider-based complementary and alternative medicine
NCI: National Cancer Institute
